# Paraganglioma of seminal vesicle and chromophobe renal cell carcinoma: a case report and literature review

**DOI:** 10.1590/S1516-31802012000100010

**Published:** 2012-02-13

**Authors:** César Augusto Alvarenga, José Manuel Lopes, João Vinagre, Paula Itagyba Paravidino, Marcelo Alvarenga, Adilson Prando, Lísias Nogueira Castilho, Paula Soares, Athanase Billis

**Affiliations:** I PhD. Pathologist, Institute of Pathology of Campinas (Private Laboratory), Campinas, São Paulo, Brazil, and Fellow at Institute of Molecular Pathology and Immunology, University of Porto (IPATIMUP), Porto, Portugal.; II PhD. Pathologist, Institute of Molecular Pathology and Immunology, University of Porto (IPATIMUP), Porto, Portugal.; III MSc. Researcher, Institute of Molecular Pathology and Immunology, University of Porto (IPATIMUP), Porto, Portugal.; IV MD. Pathologist, Institute of Pathology of Campinas (private laboratory) Campinas, São Paulo, Brazil, and Fellow of Institute of Molecular Pathology and Immunology, University of Porto (IPATIMUP) Porto, Portugal.; V PhD. Pathologist, Universidade Estadual de Campinas (Unicamp), and Pathologist, Institute of Pathology of Campinas (Private Laboratory), Campinas, São Paulo, Brazil.; VI PhD. Radiologist, Department of Radiology, Universidade Estadual de Campinas (Unicamp), and Radiologist, Hospital Vera Cruz, Campinas, São Paulo, Brazil.; VII PhD. Urologist, Department of Urology, Pontifícia Universidade Católica de Campinas (PUC-Campinas), Campinas, São Paulo, Brazil.; VIII PhD. Senior Researcher, Institute of Molecular Pathology and Immunology, University of Porto (IPATIMUP), Porto, Portugal.; IX PhD. Titular Professor, Department of Pathology, Universidade Estadual de Campinas (Unicamp), Campinas, São Paulo, Brazil.

**Keywords:** Paraganglioma, Carcinoma, renal cell, Seminal vesicles, Kidney neoplasms, Mutation, Paraganglioma, Carcinoma de células renais, Glândulas seminais, Neoplasias renais, Mutação

## Abstract

**CONTEXT:**

Extra-adrenal paragangliomas are rare tumors that have been reported in many locations, including the kidney, urethra, urinary bladder, prostate, spermatic cord, gallbladder, uterus and vagina.

**CASE REPORT:**

This report describes, for the first time to the best of our knowledge, a primary paraganglioma of the seminal vesicle occurring in a 61-year-old male. The patient presented persistent arterial hypertension and a previous diagnosis of chromophobe renal cell carcinoma. It was hypothesized that the seminal vesicle tumor could be a metastasis from the chromophobe renal cell carcinoma. Immunohistochemical characterization revealed expression of synaptophysin and chromogranin in tumor cell nests and peripheral S100 protein expression in sustentacular cells. Succinate dehydrogenase A and B-related (SDHA and SDHB) expression was present in both tumors.

**CONCLUSIONS:**

No genetic alterations to the *VHL* and *SDHB* genes were detected in either the tumor tissue or tissues adjacent to the tumor, which led us to rule out a hereditary syndrome that could explain the association between paraganglioma and chromophobe renal cell carcinoma in a patient with arterial hypertension.

## RESUMO

**CONTEXTO:**

Paragangliomas extra-adrenais são tumores raros que têm sido relatados em muitas localizações, incluindo rim, uretra, bexiga, próstata, cordão espermático, vesícula biliar, útero e vagina.

**RELATO DE CASO:**

Este relato descreve, pela primeira vez em nosso conhecimento, um paraganglioma primário da vesícula seminal ocorrendo em um paciente do sexo masculino de 61 anos de idade. O paciente apresentou hipertensão arterial persistente e um diagnóstico prévio de carcinoma de células renais cromófobo (CCRC). Foi pensado que o tumor de vesícula seminal poderia ser uma metástase do CCRC. A caracterização imunoistoquímica revelou expressão de sinaptofisina e cromogranina nos ninhos de células tumorais e expressão de proteína S100 nas células sustentaculares. Expressão de succinato de-hidrogenase A e B relacionada (SDHA e SDHB) estiveram presentes em ambos os tumores.

**CONCLUSÕES:**

Nenhuma alteração genética dos genes *VHL* e *SDHB* foi detectada nos tecidos tumorais e adjacentes ao tumor, o que nos levou a afastar uma síndrome hereditária que poderia explicar a associação entre o paraganglioma e o CCRC em um paciente com hipertensão arterial.

## INTRODUCTION

Paragangliomas are tumors of neural crest-derived endocrine cells or organs that originate in locations corresponding to the sites in which normal paraganglia occur (such as the head and neck region, vagus nerve and extra-adrenal region, i.e. the abdomen and thoracic sympathoadrenal neuroendocrine system) during development or in adulthood. The prototypical sympathetic paraganglia are the adrenal medulla and the organ of Zuckerkandl. The prototypical parasympathetic paraganglia is the carotid body. Other paraganglia are microscopic and have variable locations.^1^

Nearly 85% of paragangliomas are intra-abdominal, 12% are intrathoracic, and 3% are cervical. Paragangliomas may occur in unusual sites, including the kidneys, urethra, urinary bladder, prostate, spermatic cord, gallbladder, uterus and vagina.^2^

To the best of our knowledge, this is the first reported case of a primary paraganglioma in the seminal vesicle. An additional occurrence was the association with a chromophobe renal cell carcinoma in the same patient (Table 1).

## CASE REPORT

**Figure 1.**
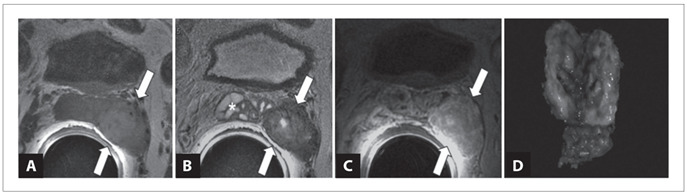
Paraganglioma of seminal vesicle seen on endorectal magnetic resonance imaging. A) T1-weighted axial image showing a slightly hyperintense solid mass in the left seminal vesicle (arrows).The presence of focal hyperintense areas within the mass is due to the hemorrhagic component of the lesion; B) T2-weighted axial image demonstrating that the mass (arrows) is predominantly hypointense. Note the normal appearance of the right seminal vesicle (*); C) Contrast enhanced, T1-weighted axial image showing that the lesion (arrows) presents a high degree of contrast enhancement. D) Macroscopic appearance: seminal vesicle with a brownish, well-circumscribed smooth nodule measuring 30 mm.

A 61-year-old obese male underwent laparoscopic partial nephrectomy due to an incidental tumor in the left kidney that was found during a work-up for hypertension. Microscopic examination of the tumor revealed that this was a chromophobe renal cell carcinoma. After one year of surveillance, a routine follow-up evaluation revealed a tumor in the left seminal vesicle. Magnetic resonance imaging (MRI) showed a well-circumscribed heterogeneous solid tumor in the left seminal vesicle measuring 32 mm across its largest dimension, with well-defined cleavage planes with the rectum and bladder walls (Figure 1). The patient then underwent laparoscopic surgical excision of the left seminal vesicle.

**Figure 2.**
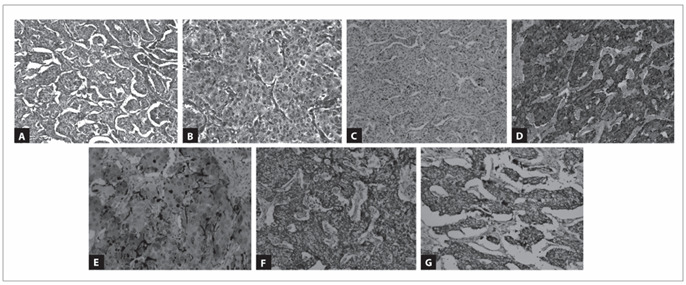
Paraganglioma of seminal vesicle: low-power (A); high-power (B); negative expression of CK7 (C); diffuse positive expression of chromogranin (D); positive expression of S100 in sustentacular cells (E); and positive expression of SDHA (F) and SDHB (G).

Grossly, the seminal vesicle measured 6 x 3 x 2 cm, and the cut surface showed a solid, well-circumscribed, brownish and smooth nodule measuring 30 mm across its largest dimension. The surgical margins were free from tumor. Microscopic examination of the tumor disclosed well-defined nests of cuboidal cells separated by vascular fibrous septa without evidence of vascular invasion, mitotic figures or necrosis. Gland-like structures were identified focally. The individual tumor cells had a large central nucleus and small to medium-sized nucleoli, and granular eosinophilic cytoplasm (Figure 2).

The diagnostic possibilities at this point included metastasis of the previous chromophobe renal cell carcinoma, adenocarcinoma of the seminal vesicle and paraganglioma. Immunohistochemical characterization was used for the differential diagnosis (antibodies summarized in Table 2). The tumor cells were immunoreactive for chromogranin and synaptophysin (Figure 2). At the periphery of the tumor nests, S100 protein-positive cells were identified, probably corresponding to sustentacular cells, in the absence of tumor keratin expression (Figure 2). Absence of immunoreactivity for keratins (AE1/AE3, CK7 and CK8/18) ruled out the hypothesis of primary or metastatic carcinoma, and supported the diagnosis of seminal vesicle paraganglioma. The Ki-67 labeling index was less than 2%. VHL and SDHB mutations were investigated using genomic DNA extracted from paraffin-embedded tumor sections (both from the seminal vesicle tumor and from the chromophobe renal cell carcinoma). No genetic alterations were found either in the *VHL* or in the *SDHB* genes.

Thorough imaging analysis showed that there was no tumor elsewhere, which therefore reinforced the diagnosis of primary seminal vesicle paraganglioma. The patient is still alive after 14 months of follow-up and his blood pressure is under control.

**Table 1.** Published papers relating to seminal vesicle paraganglioma associated with chromophobe renal cell carcinoma, according to database (March 16, 2011).

Database	Search strategy	Results
PubMed	“Seminal vesicle paraganglioma”	No case reports or reviews of the literature
	“Chromophobe renal cell carcinoma AND seminal vesicle paraganglioma”	No case reports or reviews of the literature
Lilacs	“Seminal vesicle paraganglioma”	0 reports
	“Chromophobe renal cell carcinoma AND seminal vesicle paraganglioma”	0 reports
Cochrane Library	“Seminal vesicle paraganglioma”	0 reports
	“Chromophobe renal cell carcinoma AND seminal vesicle paraganglioma”	0 reports

**Table 2.** List of antibodies used in the immunohistochemistry study

Antigen	Origin	Clone	Dilution
CK7	Zymed	OV-TL12/30	1/50
CD10	Novocastra	NCL-L_CD10-270	1/10
Vimentin	Dako	V9	1/150
CK8/18	Cell Marque	B22.1 & B23.1	1/50
AE1/AE3	Zymed	AE1/AE3	1/50
Chromogranin	Neomarkers	SP12	1/200
Synaptophysin	Cell Marque	Polyclonal	1/300
S100	Dako	S100	1/2000
Ki-67	Neomarkers	Ki-67 (SP6)	1/300
SDHA	Mitosciences Inc	2E3GC12FB2AE2	1/1250
SDHB	Mitosciences Inc	21A11AE7	1/600

## DISCUSSION

Parasympathetic ganglia-derived tumors are found almost exclusively in the neck and skull base and arise within the carotid body and jugulotympanic glomus.^3^ In a review of 236 patients with paragangliomas, Erickson et al.^4^ found that 69% of paragangliomas were found in the head and neck region and that the majority were parasympathetic in origin. In contrast, sympathetic paragangliomas, also known as extra-adrenal pheochromocytomas, arise outside of the adrenal gland and can be found anywhere along the sympathetic chain from the base of the skull and neck (5% of cases) to the bladder and prostate gland (10%).^3^ About 90% of sympathetic paragangliomas occur in adults and 90% of these are intra-adrenal (pheochromocytomas). About half of the extra-adrenal sympathetic tumors arise in the organs of Zuckerkandl, and most of the remainder in the retroperitoneum. There is equal distribution between the sexes, except in children and in patients with thoracic tumors, among whom males are reported to be more commonly affected.^5^

There are some reports in the urology literature about paragangliomas located in the prostate, bladder and even the paratesticular region (six cases).^2^ The histogenesis of paragangliomas of the spermatic cord is unknown, although it has been speculated that paraganglion nests in the spermatic cord may be secondary to dysgenesis during embryogenesis.^2^ The present study is the first report on primary paraganglioma of the seminal vesicle. Its origin can be explained in the same way as for the spermatic cord, because the cells that give rise to the seminal vesicle originate in the caudal Wolffian duct and urogenital sinus prostate.^6^

Paragangliomas and pheochromocytomas can occur sporadically or in the context of several inherited tumor syndromes, including multiple endocrine neoplasia type 2 (MEN2, with *RET* germline mutations), von Hippel-Lindau (VHL) disease (caused by germline mutations in the *VHL* gene), neurofibromatosis type 1 (NF1, with *NF1* gene germline mutations) and pheochromocytoma-paraganglioma syndrome. The latter syndrome is the most frequent hereditary condition with manifestations of paragangliomas, and is caused by mutations in the *SDHB*, *SDHC* or *SDHD* genes.^3^^,^^7^ The syndrome is characterized by familial occurrence of pheochromocytoma or paragangliomas, usually at a young age, and often with multifocal disease. In the setting of *SDHB* mutations, the tumors show greater risk of recurrence and higher frequency of malignancy.^3^^,^^7^

In view of the rare association of paraganglioma and chromophobe renal cell carcinoma in our patient (Table 1), the presence of *VHL* and *SDHB* mutations was investigated using genomic DNA extracted from paraffin-embedded tumor sections (both from the seminal vesicle tumor and from the chromophobe renal carcinoma). No genetic alterations were found either in the *VHL* or in the *SDHB* genes.

## CONCLUSIONS

In conclusion, this is the first report of a primary paraganglioma in the seminal vesicle, with an additional association with chromophobe renal cell carcinoma (Table 1). Paragangliomas may occur sporadically or in hereditary syndromes associated or caused by *VHL* and *SDHB* mutations. Using genomic DNA extracted from paraffin-embedded sections through the tumors, no genetic alterations were found. Hence, the diagnosis of a sporadic primary tumor of the seminal vesicle was favored.
